# Philadelphia County Medical Association

**Published:** 1880-01

**Authors:** 


### PHILADELPHIA COUNTY MEDICAL ASSOCIATION.

A conversational meeting was held at the hall of the College of Physicians, Philadelphia, November 12, 1879, Prof. Henry H. Smith, President of the Society, in the chair.

Dr. J. A. McFerran read a paper entitled "Tactus Eruditus" (withdrawn by the author.)

Dr. E. T. Bruen reported some unusual cases of anasarca.

Dr. Frederick P. Henry inquired what process had been adopted for counting the blood-corpuscles in attempting to decide the question of anæmia. He understood the lecturer to state that, in the only case in which a count of the corpuscles was made, their number was found to be 4,750,000 per cubic millimetre. Whether this is evidence of the existence of anæmia or not would depend, to a great extent, upon the kind of instrument employed. With Malassez's instrument such a count would be above the normal average, while with Gower's or Nachet's it would be somewhat below it. It is impossible to decide in some cases, from the general appearance of the patient, whether he be anæmic or not. He recalled a case that he had recently examined with Dr. Nancrede, in which an examination of the blood showed that there were only about 3,000,000 red cells per cubic millimetre, while there was an enormous increase in the white cells, their proportion to the red being as two to three. From an ordinary observation no one would have supposed the patient to be anæmic, his face being full, and his cheeks and lips quite red.

He wished also to call attention to the fact that, in the cases just reported, there was a history of intermittent fever, and that the most acute anæmias, with the exception of that form produced by loss of blood, are caused by fevers. The rapid destruction of red blood-cells in febrile conditions is indicated by an increase in the amount of urine pigment which, in common with all the coloring-matters of the body, is derived from the red cells. According to Wagner, there is a twenty-fold increase in the amount of urine pigment in fever; which statement, although vague (fever being a relative term,) is sufficient to prove that the increase in all fevers is very great. Dr. Henry also called attention to the fact that iron had entered into the treatment of all these cases, and, taking this fact in connection with their previous history, he was hardly convinced of the non-existence in them of anæmia.

Dr. Henry, in reply to a question, stated that he would explain the presence of red lips in an anæmic patient by the supposition of an uneven distribution of the blood, as it is well known that a poor quality of the blood often coexists with frequent flushing of the face.

Dr. Bruen said that he held in his hand a copy of Nachet's directions for the use of his instrument, in which the average number of red globules is set down at 5,000,-000 per cubic millimetre. He would defer to the greater experience of Drs. Henry and Nancrede, but a series of five observations upon healthy individuals showed that in three of them the number of cells was below five millions, even to the extent of two or three hundred.

While the cases reported may have had a slight reduction in the number of the red cellular elements of the blood, there was not sufficient anæmia to account for the dropsy; and he desired to call the attention of the society to the explanation to which his own mind had been directed by a study of the subject and a few similar reported cases, viz., a diminished vaso-motor tonus in cases where the blood-crasis was not sufficiently at fault to account for the transudation. He believed that this pathology of dropsy had been neglected.

He had used the iron in these cases rather for its effect upon the blood-vessels than for its power of restoring to the red blood-disks the material necessary for their functional activity as oxiding and, therefore, eliminating agents.

He did not think that the iron in his cases had been a very active agent in the absorption, though in its place an excellent remedy.

Dr. Charles B. Nancrede recalled the fact that the blood-cells in the case referred to by Dr. Henry were only a little over two millions, while the man's appearance was decidedly that of health. The testimony of observers who have used Nachet's instrument is that the average in health is above five millions. This is the opinion of Dr. Keyes also. In referring to the cases whose blood was examined by Dr. Bruen, he said that if they were resident physicians he would not consider that they could be properly taken as instances of healthy individuals. The confinement and the hospital air tend to reduce their physical condition below the standard.

NEW DIETETIC PREPARATIONS.

Dr. M. O'Hara asked the attention of the members of the Society to some specimens of dietetic preparations for the sick that he had found very useful. They are made by the Health Food Company, and consist of mixed cereals, containing a large proportion of gluten, as well as the amylaceous constituents. In one specimen for diabetic diet the starch had been removed. The cereal coffee he had found very useful in dyspepsia. The other preparations make very nourishing dishes for the sick.

In young children he prefers Horlick's Sugar of Malt where there is defective nutrition.

Dr. Carl Seiler said that in the northern part of Germany a preparation similar to this cereal coffee is made, and is generally used as a substitute for coffee. In regard to the Sugar of Malt, he reported that he had used it in a number of cases with good results in the convalescence from typhoid, in phthsis, and in other wasting diseases. He prefers it to cod-liver oil. He did not regard it simply as a food, for he had found that, in overdoses, it produced nervous symptoms—such as dizziness and tinnitus—similar to quinine.

Dr. Wm. B. Atkinson recommended Keasby & Mattison's Infant's Food: as a restorative he had used it with excellent results. He would prefer Mellin's food to Horlick's, but considered them almost identical in composition and manufacture.

Dr. Albert H. Smith reported that he had obtained very good results from the use of Horlick's Sugar of Malt, and his Infant's Food, especially with strumous and rickety children. He preferred the malt in this granulated condition, because the children take it more readily.—*Phil. Med. Times.*

